# Dietary Walnut Supplementation Alters Mucosal Metabolite Profiles During DSS-Induced Colonic Ulceration

**DOI:** 10.3390/nu11051118

**Published:** 2019-05-20

**Authors:** Masako Nakanishi, Alyssa Matz, Cory Klemashevich, Daniel W. Rosenberg

**Affiliations:** 1 Center for Molecular Oncology, University of Connecticut Health, Farmington, CT 06030, USA; nakanishi@uchc.edu(M.N.); alyssa.matz@uconn.edu (A.Z.); 2 Integrated Metabolomics Analysis Core, Texas A&M University, College Station, TX 77843, USA; Klem24@tamu.edu

**Keywords:** walnuts, omega-3 fatty acids, lipid metabolites, inflammation, Inflammatory Bowel Disease, ulcerative colitis

## Abstract

Walnuts contain a complex array of natural compounds and phytochemicals that exhibit a wide range of health benefits, including protection against inflammation and colon cancer. In this study, we assess the effects of dietary supplementation with walnuts on colonic mucosal injury induced in mice by the ulcerogenic agent, dextran sodium sulfate (DSS). C57Bl/6J mice were started on the Total Western Diet supplemented with freshly-ground whole walnuts (0, 3.5, 7 and 14% g/kg) 2 weeks prior to a 5-day DSS treatment and walnut diets were continued throughout the entire experimental period. Mice were examined at 2 days or 10 days after withdrawal of DSS. In a separate study, a discovery-based metabolite profiling analysis using liquid chromatography tandem mass spectrometry (LC-MS/MS) was performed on fecal samples and colonic mucosa following two weeks of walnut supplementation. Dietary walnut supplementation showed significant effects in the 10-day post-DSS recovery-phase study, in which the extent of ulceration was significantly reduced (7.5% vs. 0.3%, *p* < 0.05) with 14% walnuts. In the metabolite-profiling analysis, walnuts caused a significant increase in several polyunsaturated fatty acids (PUFAs), including docosahexaenoic acid (DHA) and 9-oxo-10(E),12(E)-octadecadienoic acid (9-oxoODA), as well as kynurenic acid. In colon tissue samples, walnuts caused a significant increase in the levels of S-adenosylhomocysteine (SAH) and betaine, important components of fatty acid β-oxidation. These metabolite changes may contribute in part to the observed protection against DSS-induced inflammatory tissue injury.

## 1. Introduction

Dietary supplementation with nuts has shown a variety of health benefits. Among the tree nuts, walnuts (*Juglans regia*) contain the highest levels of the omega-3 fatty acid, namely alpha-linoleic acid (ALA), with the most favorable ratio of omega-3:omega-6 fatty acids (1:4.2) [1]. Walnuts also contain a large number of phytochemicals, including phenolic antioxidants, and high levels of nutrients with beneficial properties to guard against a variety of diseases, including heart disease, diabetes, neurological disorders, inflammation and cancer [1,2,3]. Moreover, walnuts are a rich source of fiber (up to 6.4%), which has been shown to support the maintenance of disease remission in patients suffering from inflammatory bowel disease (IBD) [4].

IBD, encompassing both Crohn’s disease (CD) and ulcerative colitis (UC), are chronic intestinal inflammatory disorders of largely unknown etiology [5]. IBD is typically characterized by alternating periods of clinical remission and disease flare-ups [6]. As recently reviewed [7], diet clearly plays an important role in IBD progression. A variety of dietary strategies have been proposed for IBD patients, many of them considered ’fad’ diets and often not sufficiently controlled for affording clinical efficacy [8]. Furthermore, many of these restricted diets that are given during remission, if not properly maintained, may ultimately contribute to nutritional deficiencies in carbohydrates, monounsaturated fatty acid (MUFAs), fiber, calcium and vitamins C, D, E and K [9]. However, epidemiological data have demonstrated that a high intake of total fats, polyunsaturated fatty acids (PUFAs) and red meat are associated with increased risk of developing active CD and UC [10]. Alternatively, diets enriched in fiber and plant-based foods, including fruits, vegetables and nuts, have shown a preventive benefit during remission [7,10].

The following study design is based upon the reported anti-inflammatory properties of walnuts [11,12,13]. Using a preclinical mouse model of UC, we have examined the potential health benefits of whole walnuts added to a Total Western Diet (TWD) [14] on the extent of intestinal injury following exposure of mice to the ulcerogenic agent, DSS. We have examined how walnut consumption, starting prior to DSS exposure and continuing throughout the entire disease course, may influence the extent of intestinal inflammation during both the acute and recovery phases of the experimentally-induced disease. To gain further insight into how walnut consumption may ’condition’ the colonic mucosa towards a protective state, we have conducted a discovery-based metabolomic profiling analysis on both fecal samples and colonic mucosa obtained from mice maintained for two weeks on a walnut-supplemented diet. This analysis has identified significant increases in a number of lumenal and tissue metabolites, several of which may contribute to the observed protection from ulcerogenic injury observed in this study. 

## 2. Materials and Methods 

### 2.1. Animal Treatment

C57BL/6 (B6) mice were fed the TWD ([14]: Envigo, Madison, WI, USA) supplemented with freshly ground whole walnuts throughout the entire experimental period. Fixed amounts of the diet were given each week based on an average daily food intake of 3.5 g/day/mouse. The amount of walnuts added to the experimental diet was determined based on previous preclinical studies from our laboratory and others [2,15,16]. These concentrations are consistent with a human dietary consumption of 56.6 g (2 oz) of walnuts per day, amounting to ~18% percent of total caloric intake based on 2000 calories per day [1]. Mice were maintained on a standard rodent diet (Teklad Global 18% protein rodent diet, Envigo) until 7 weeks of age, then switched to the TWD supplemented with 0, 3.5, 7 or 14% walnut by weight, which is equivalent to 0, 5.2, 10.5 or 21.4% of total energy supplied from walnuts [2]. The relative dietary content of the individual fat sources was reduced proportionately to compensate for the addition of walnuts (Supplementary Table S1). Walnuts were finely ground and added to the diet, which was freshly prepared each week. 

Two weeks after the start of the walnut diet, mice were divided into two separate studies. In Study 1 (’acute phase’), a total of 40 mice were administered 2% DSS in the drinking water for five days and then returned to regular drinking water. These mice were then sacrificed at two days after withdrawal from DSS. A single mouse each in the 0% and 3.5% walnut groups died immediately after DSS treatment and are not included in the analyses. In Study 2 (’recovery phase’), a total of 40 mice were administered 1% DSS in the drinking water for five days and then returned to regular drinking water. These mice were then sacrificed at ten days after withdrawal from DSS. Even at 1% DSS, 4 mice from the 0% group and one mouse from 3.5% walnut group did not survive the recovery phase and are not included in the analyses. 

For the walnut metabolomics study, mice were fed the TWD diet containing either 0% or 14% walnuts for two weeks. Fecal samples from each mouse were collected separately at days 0 and 14 and stored at −80 °C until analysis. Body weights (*b.w*.) were recorded once per week. Both male and female mice were used in the study, and the data are pooled for statistical analyses. All mice were maintained in a light-cycled, temperature-controlled room and allowed free access to drinking water and diet. All animal experiments were conducted under an animal protocol (101369-0519) approved on May 31, 2016 by the Center for Comparative Medicine (CCM) at the University of Connecticut Health Center, and were performed in strict accordance with all Institutional Animal Care and Use Committee (IACUC) guidelines.

### 2.2. Tissue Processing and Analyses of Ulceration and Restitution 

At sacrifice, colons and spleens were harvested from each mouse. Spleens were weighed and colons were immediately flushed with ice-cold PBS and slit-open longitudinally. Colons were divided longitudinally into the following two sections: formalin-fixed and paraffin-embedded (FFPE) for histologic evaluation and snap-frozen at −80 °C for metabolite analysis. FFPE tissues were sectioned at 5 μm and hematoxylin & eosin (H&E)-stained for histological analysis. The percentage of colonic ulceration and restitution was calculated by measuring the total length of ulcerated or restituted areas across the entire length of colon [17]. 

### 2.3. Measurement of Fecal and Tissue Metabolites

For the metabolite analyses, 3 fecal samples per group (days 0 and 14), and 10 tissue samples per group (0 and 14%) were used. Samples were extracted with a methanol:chloroform:water-based extraction method with a minor modification [18]. Briefly, 800 μL of ice-cold methanol:chloroform (1:1, *v*:*v*) was added to each sample and homogenized on a Precyllys 24 tissue homogenizer (Bertin, Rockville, MD, USA). The supernatant was collected and samples were further homogenized a second time with 800 μL of ice-cold methanol:water. The supernatants were pooled and filtered through a 0.2 um nylon filter and then passed through a 3-kDa protein filter (Merck Millipore, Burlington, MA, USA). After adding 600 μL of ice-cold water, samples were vortexed and centrifuged to collect the aqueous upper phase, which was passed through a 3-kDa cut-off column (Thermo Scientific, Waltham, MA, USA) and the flow-through was collected for subsequent analysis.

Untargeted liquid chromatography high-resolution accurate mass spectrometry (LC-HRAM) analysis was performed on a Q Exactive^TM^ Plus Hybrid Quadripole-Orbitrap Mass Spectrometer (Thermo Scientific) coupled to a binary pump ultra-high-performance liquid chromatography (UHPLC) (UltiMate 3000, Thermo Scientific). Full MS spectra were obtained at 70,000 resolution (200 m/z) with a scan range of 50–750 m/z. Full MS followed by ddMS2 scans were obtained at 35,000 resolution (MS1) and 17,500 resolution (MS2). Samples were maintained at 4 °C prior to injection. The injection volume was 10 µL. Chromatographic separation was achieved on a Synergi Fusion 4 µm, 150 mm × 2 mm reverse phase column (Phenomenex, Torrance, CA) maintained at 30 °C using a solvent gradient method with solvent A (0.1% formic acid in water) and solvent B (0.1% formic acid in methanol). The gradient method used was as follows: 0–5 min (10% B to 40% B), 5–7 min (40% B to 95% B), 7–9 min (95% B), 9–9.1 min (95% B to 10% B) and 9.1–13 min (10% B). The flow rate was set to 0.4 mL min^−1^. Sample acquisition was performed Xcalibur (Thermo Scientific). Data analysis was performed with Compound Discoverer 2.1 (Thermo Scientific). 

Compounds annotated in Compound Discoverer were defined as a mass at a specific retention time. Compounds were putatively identified (named) with annotation criteria from the mzCloud database or the Biocyc, HMDB and KEGG databases within ChemSpider. The mzCloud database annotation was utilized for putative identifications, except in the case where authentic standards were used to validate compounds of interest [19].

### 2.4. Statistical Analyses

Statistical analyses were performed using GraphPad Prism V software (GraphPad Software, Inc., La Jolla, CA, USA). Data are reported as the means ± standard error of the mean (SEM). *p-*values were calculated by the Student’s *t*-test, one-way analysis of variance (ANOVA) with Bonferroni’s multiple comparison tests or linear-regression analyses where appropriate, as indicated in the Legends to Figures. A *p*-value less than 0.05 was considered statistically significant. For metabolomics analyses, *p*-values for the treatment group were calculated by the Tukey’s honestly significant difference (HSD) test (*post-hoc*) after an ANOVA test, adjusted by using the Benjamini-Hochberg correction for a false-discovery rate. 

## 3. Results

### 3.1. Dietary Walnut Supplementation Protects Mice From DSS-Induced Intestinal Injury

#### 3.1.1. Effects of Walnut Supplementation in Acute Phase of DSS-Induced Colitis

**Figure 1:**
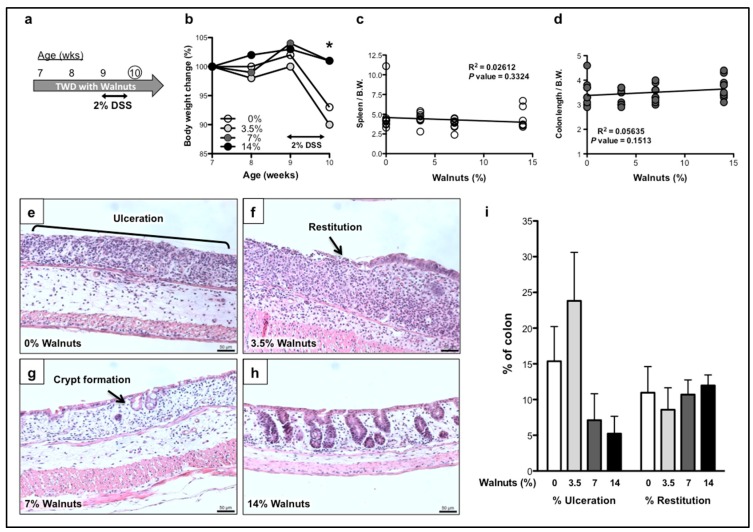
Effects of walnut supplementation on DSS-induced acute colitis. (**a**) Experimental design for the acute phase study. TWD diet supplemented with 0, 3.5, 7 or 14% walnuts was started two weeks prior to the administration of 2% DSS. Mice were sacrificed 2 days after the withdrawal of DSS. The effects of walnut supplementation were examined on the following clinical endpoints: body weight change (**b**), linear-regression analyses of spleen weight (**c**) and colon length (**d**). DSS-induced colonic ulceration (**e**), with signs of restitution (**f**) and crypt formation (**g**). There was significantly less ulceration with 14% walnuts (**h**). Extent of colonic ulceration and restitution were scored as a percent of the entire length of the colon for each group (**i**). Bars represent the means ± SEM *n* = 9 for 0%, *n* = 9 for 3.5% and *n* = 10 for 7% and 14%. * One-way ANOVA with Bonferroni’s multiple comparison tests, *p* < 0.05.

In the first study, we examined the effects of walnut supplementation on the extent of colonic injury during the acute phase of DSS-induced colitis. Mice were fed TWD diet supplemented with 0, 3.5, 7% or 14% walnuts, starting at two weeks prior to 2% DSS treatment and maintained throughout the experimental period (Figure 1a). Mice were sacrificed at 2 days after the withdrawal of DSS. As shown in Figure 1b, mice fed the two highest concentrations of walnuts maintained normal body weight throughout the DSS treatment period, compared to the 0 and 3.5% groups. Spleen weights and colon lengths showed no significant correlation with walnut supplementation by linear-regression analyses (Figure 1c,d). 

As shown in Figure 1e, histological examination of the colons from DSS-treated control mice showed large areas of mucosal ulceration (~15% of the entire colon, Figure 1i) with no obvious remaining cryptal structure evident during the acute phase of the disease. Although there were still large areas of colonic ulceration present in mice ingesting 3.5% walnuts, a prominent leading edge of columnar epithelial cells was readily apparent, indicating the restorative process of colonic restitution (Figure 1f). At the higher walnut concentrations (7 and 14%), re-epithelialized areas of colonic mucosa were clearly visible, generally comprised of a single layer of epithelial cells (restituted areas) and often with clear evidence of new crypt formation (Figure 1g), an outcome that was more frequently observed in the 14% walnut group (Figure 1h). There was an approximate 3-fold reduction in the overall percentage of colonic ulceration in mice fed either 7% or 14% walnuts, although the lowest concentration of walnuts (3.5%) modestly increased the percent of ulceration (Figure 1i). Linear-regression analysis showed a significant walnut concentration-dependent reduction in the extent of ulceration (*p* = 0.0470, Supplementary Figure S1a). However, there were no statistically significant differences in the total areas of colonic restitution at two days after DSS treatment (Figure 1i and Supplementary Figure S1b).

#### 3.1.2. Effects of Walnut Supplementation in Recovery Phase of DSS-Induced Colitis

**Figure 2:**
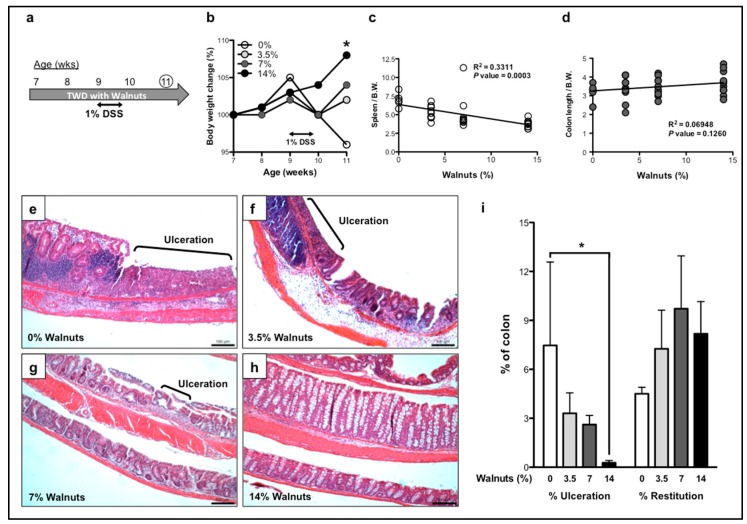
Effects of walnut supplementation on the recovery from dextran sodium sulfate (DSS)-induced colitis. (**a**) Experimental design for the recovery phase study. Total Western Diet (TWD) supplemented with 0, 3.5, 7 or 14% walnuts was started two weeks prior to the administration of 1% DSS. Mice were sacrificed 10 days after the withdrawal of DSS. The effects of walnut supplementation were examined on the following clinical endpoints: body weight change (**b**), linear-regression analyses of spleen weight (**c**) and colon length (**d**). The effects of walnut concentration on recovery from DSS induced colitis is shown for 0% (**e**) 3.5% (**f**) 7% (**g**) and 14% walnuts (**h**). The extent of ulceration and restitution were scored as the percent of the entire length of colon for each group (**i**). Bars represent the means ± SEM. *n* = 6 for 0%, *n* = 9 for 3.5% and *n* = 10 for 7% and 14%. * One-way ANOVA with Bonferroni’s multiple comparison tests, *p* < 0.05.

In the second study, we examined the effects of walnut consumption on the recovery phase of the experimentally-induced disease. As shown in Figure 2a, mice were placed on walnut-containing TWD for two weeks before being administered 1% DSS. Mice were then maintained on the walnut-containing diet for an additional 10 days after withdrawal from 1% DSS. The walnut-fed mice showed a more rapid recovery in body weight that was associated with the walnut concentration (Figure 2b). In addition, there was a significant reduction in spleen weights (*p* = 0.0003, Figure 2c) and an increasing, but non-significant trend in colon lengths associated with walnut supplementation (*p* = 0.1260, Figure 2d). 

During the recovery phase, there was a striking, walnut concentration-dependent reduction in total ulcerated areas of the colonic mucosa. As shown in Figure 2e,i, mice that did not receive walnuts displayed large and extensive areas of colonic ulceration at 10 days post-DSS. The extent of colonic ulceration decreased proportionately with the concentrations of walnut supplementation (Figure 2f–h). In fact, at the highest concentration of walnuts ingested (14%), damaged epithelium accounted for less than 1% of the total mucosal area, a remarkable degree of mucosal repair (Figure 2h,i, Supplementary Figure S1c). In addition, the walnut-fed mice showed an increase in epithelial restitution when compared to the dietary control group, however it didn’t reach statistical significance (Figure 2i and Supplementary Figure S1d). These results provide experimental evidence that walnut supplementation, particularly at the highest concentrations, can protect the colonic mucosa against DSS-induced colitis.

### 3.2. Walnut Consumption Alters Fecal Metabolite Composition 

We postulate that the protection afforded to the colonic mucosa by walnut ingestion results from the ability of walnuts to somehow ’condition’ the colonic mucosa, creating an inflammation-suppressive microenvironment that protects mice against subsequent DSS-induced ulcerogenic injury. To determine whether walnut supplementation may adapt the colonic mucosa to subsequent injury by modifying the composition of luminal metabolites, we performed a discovery-based metabolite profiling analysis. Metabolite profiles present in fecal samples were examined by LC-HRAM analysis, before and after feeding a diet containing 14% walnuts for two weeks. Among the 3200 compounds that were present within the fecal samples, 186 compounds matched those within the mzCloud database at a match factor of 50 [19], in which 154 compounds were increased and 32 compounds were decreased. Compounds with a *p*-value less than 0.1 are shown in Table 1. While only one compound showed a significant reduction (Dodecyltrimethylammonium; C15H33N; 0.53-fold; *p* = 0.0310), there were 13 positively identified metabolites that were significantly increased upon walnut supplementation (Table 1). 

As expected, walnut consumption increased the fecal levels of both omega-3 and omega-6 fatty acids, including docosahexaenoic acid (DHA; C22H32O2; 8.33-fold; *p* = 0.009) and arachidonic acid (AA; C20H32O2; 7.42-fold; *p* = 0.0283), respectively. Several metabolites of linoleic acid were also markedly elevated, including 9-oxo-10(E),12(E)-octadecadienoic acid (9-oxoODA; C18H30O2; 29.29-fold; *p* = 0.0001), which has been reported to activate peroxisome proliferator-activated receptor-α (PPARα), a key regulator of lipid metabolism [20]. There was also a significant increase in several tryptophan metabolites, including kynurenic acid (6.4-fold; *p* = 0.0042) (C10H7NO3), a product of the kynurenine pathway of tryptophan metabolism with potent anti-inflammatory and immunosuppressive properties [21]. An additional tryptophan metabolite, N-acetyl serotonin, was also elevated (2.4-fold, *p* = 0.022). The presence of these metabolites is not unexpected since walnuts are a good source of tryptophan, with one serving containing 318 mg. Several other amino acids were also modestly elevated in the fecal samples, including D-(+)-proline (C5H9NO2; 3.42-fold; *p* = 0.0078) and L-methionine sulfoxide (C5H11NO3S; 1.77-fold; *p* = 0.0898). D-Proline is an isomer of naturally occurring L-Proline, generated by a bacterial enzyme, proline racemase [22]. In fact, proline-rich antimicrobial peptides have been shown to interfere with protein synthesis in pathogenic microorganisms [23].

Finally, experimental conditions were optimized using authentic standards to determine the levels of the ellagitannin hydrolysis product, ellagic acid, a potent antioxidant that is present in high concentrations in walnuts [24,25]. Although walnut-induced changes to the fecal levels of ellagic acid (C14H6O8) did not reach statistical significance, the concentrations were increased by approximately 8.25-fold (*p* = 0.0883) by 14% walnut supplementation (Table 1). 

### 3.3. Accumulation of Phytochemicals in Colon Tissue Associated with Walnut Supplementation 

To examine the direct effects of walnut supplementation on the colonic mucosa, tissue extracts were prepared from the colons of mice maintained on either 0% or 14% walnuts for two weeks and then examined by LC-HRAM analysis. Among a total of 987 metabolites, 95 compounds matched with those in the mzCloud database at a match factor of 50 [19]. There were 62 compounds that showed an increase, while 33 compounds were decreased following walnut consumption. As shown in Table 2, of the group of compounds that were putatively identified, each wincreased to a relatively similar extent, with *p*-values less than 0.2. Similar to the results obtained with the fecal samples, only a single compound was found to be decreased by walnut supplementation (Bis-methylbenzylidene-sorbitol; C22H26O6; 0.79-fold; *p* = 0.0069). One interesting compound that was increased in the colon tissue was olomoucine (C15H18N6O; 1.73-fold; *p* = 0.0098), previously identified as a competitive inhibitor of the cyclin-dependent kinases (CDKs), an important family of regulators of the cell cycle [26]. Also increased within the tissue were several compounds that are involved in one-carbon metabolism. For example, S-adenosylhomocysteine (SAH; C14H20N6O5S), an amino acid derivative that can give rise to homocysteine, leading to the generation of cysteine and adenosine, modestly increased in colon tissue (1.50-fold; *p* = 0.0685). There was also an increase in betaine (C5H11NO2; 1.26-fold; *p* = 0.1157), an osmolyte and methyl donor that is commonly present within a variety of food types and is directly involved in the synthesis of methionine from homocysteine [27]. In addition, we found an increase in adenosine 5’-monophosphate (AMP; C10H14N5O7P), a hydrolysis product of adenosine diphosphate (ADP) and adenosine triphosphate (ATP) that was slightly increased (1.67-fold; *p* = 0.0951). Overall, these results indicate that a number of fundamental metabolic processes may be stimulated by walnut supplementation. These changes, although not reaching statistical significance, were clearly trending higher in the walnut supplementation group.

## 4. Discussion

Walnuts contain a complex array of natural compounds and phytochemicals with a broad range of health benefits, including protection against some forms of cancer and inflammation [1,28]. In this study, we demonstrate for the first time that dietary supplementation with walnuts protects the colonic epithelium against mucosal injury induced by the ulcerogenic agent, DSS. While even the lowest concentration of walnuts (3.5%) present in the diet showed a moderate reduction in the extent of colonic ulceration, the protection by walnuts was most pronounced during the recovery phase. As shown in Figure 2h, at ten days after DSS treatment, mice consuming a diet containing 14% walnuts for four weeks showed almost no evidence of remaining colonic ulceration. Given the likelihood that adjacent undamaged epithelial cells are critical for initiating the wound-healing process [29], we believe that walnut-fed mice have sustained less initial mucosal damage. As a consequence of the attenuated destruction of the colonic mucosa, a more rapid and robust recovery from the initial damage is possible. Based upon these observations, we believe that walnut supplementation given prior to DSS treatment may somehow adapt the colonic mucosa towards an inflammation-suppressive microenvironment that affords significant protection against DSS-induced inflammatory insult. 

The anti-inflammatory properties of whole walnuts and walnut extracts have been demonstrated in several preclinical inflammatory disease models. For example, dietary supplementation with whole walnuts resulted in a significant decrease in macrophage infiltration and suppression of pro-inflammatory gene expression (tumor necrosis factor; TNF-α, interleukin-6; IL-6 and IL-10) in a mouse model of high-fat diet (HFD)-induced fatty liver disease [12]. Similarly, lung injury induced by bleomycin (BLM) was significantly reduced with dietary walnuts, accompanied by a decline in tissue biomarkers of oxidative stress and the alveolar macrophage inflammatory response [30]. More recently, Koh et al. [31] have shown that treatment with a walnut phenolic extract (WPE) attenuated DSS-induced acute colitis, while inhibiting the production of several key pro-inflammatory cytokines, including IL-8 and IL-1α. In addition, WPE inhibited NF-κB DNA binding activity. Our results are consistent with these earlier studies indicating that walnuts can exert a positive effect on lipid metabolism. As a consequence, walnuts enhance antioxidant activity in the tissue, which will ultimately contribute to the observed suppression against mucosal ulceration.

The beneficial effects of walnuts may be attributed in part to the actions of the omega-3 fatty acids. It is well-established that the primary omega-3 fatty acid found in walnuts, alpha-linoleic acid (ALA), and its metabolites, EPA and docosahexaenoic acid (DHA), possess potent anti-inflammatory properties [32]. In fact, in the present study we found a significant increase in the levels of DHA in fecal samples collected from mice ingesting walnuts (Table 1). Among its many actions, DHA provides an important substrate for the pro-resolving mediators, (SPMs), including the resolvins, protectins and maresins, that are synthesized in the tissue during the acute phase of the inflammatory response [33]. These bioactive lipids facilitate the clearing of inflammatory cells and mediators, thereby enhancing resolution of tissue injury [33]. Although this class of bioactive lipid was not identified at significant levels within the tissue, it is possible that biologically relevant changes to these important lipid mediators have occurred at levels that are below our ability to detect. Thus, future studies with more sensitive analytical capabilities are warranted to evaluate these possibilities.

In addition to the omega-3 metabolites, our data suggest that omega-6 fatty acids present in walnuts may also contribute to protection. In fact, our study is the first to show a significant increase in the levels of the omega-6 metabolite, 9-oxoODA, upon walnut supplementation (Table 1). 9-oxoODA is abundantly present in tomatoes, a food that is known to improve overall lipid metabolism [34]. It has also been shown that 9-oxoODA under some conditions can increase PPARα expression in mouse primary hepatocytes, which in turn causes a decrease in triglyceride accumulation [20]. PPARα is a potent metabolic regulator that can directly modulate inflammatory signaling [35]. In fact, one of the most important roles of PPARα is its ability to inhibit NF-κB activity [36], a potential mechanism associated with the property of walnuts extract as an anti-inflammatory agent [31]. 

Another important property of PPARα is its ability to transcriptionally regulate the expression of a panel of genes involved in fatty acid metabolism, including carnitine-palmitoyl transferase-1 (CPT1) and acyl-CoA synthase (ACS) [37]. Interestingly, we found that several key metabolites related to fatty acid β-oxidation were increased within the colonic mucosa, including SAH and betaine (Table 2). Fatty acid β-oxidation is a multistep enzymatic process responsible for the catabolism of fatty acids, generating cellular energy in the form of ATP [38]. As reviewed by Rinaldo et al. [39], disorders of fatty-acid metabolism caused by loss of function mutations to key pathway components, such as the fatty acid transporter (organic cation transporters novels; OCTNs and carnitine-acylcarnitine translocases; CACTs) and the acyl-coenzyme A dehydrogenases (very long-chain acyl-CoA dehydrogenases; VLCADs and short-chain acyl-CoA dehydrogenases SCADs), result in pathological conditions, including liver failure and cardiac and skeletal myopathy. In the intestines, mutations in the carnitine transporter genes, OCTN1 and OCTN2, have been shown to be associated with Crohn’s disease and mice deficient in OCTN2 have been reported to develop spontaneous colonic atrophy and inflammatory changes [40]. Moreover, a four-week treatment with propionyl-L-carnitine (PLC), an ester of L-carnitine required for the mitochondrial transport of fatty acids, restores endothelial β-oxidation and function, as well as reducing intestinal mucosal inflammation in patients with mild-to-moderate UC [41]. In fact, omega-3 fatty acids have been shown to induce fatty acid β-oxidation, causing reduced levels of triglycerides, providing a clinical treatment option for hypertriglyceridemia [42]. There is also a study by Shimoda et al. [43], which showed that a polyphenol-rich extract from walnuts exhibited hypotriglyceridemic effects by enhancing peroxisomal fatty acid β-oxidation in the liver. Based on our data and others, we speculate that regular intake of walnuts increases the levels of DHA and 9-oxoODA, thereby enhancing lipid metabolism, an effect that may prevent lipid-mediated inflammation associated with IBD. 

The significant increase in the tryptophan metabolite, kynurenic acid, may have important implications regarding the protection to the colon afforded by walnuts. In a recent comprehensive review by Wirthgen et al. [21], the remarkably broad scope of biological activity exerted by kynurenic acid is discussed. As a product of the kynurenine pathway of tryptophan metabolism, kynurenic acid exerts potent anti-inflammatory and immunosuppressive properties via its ability to act as a ligand of G protein-coupled receptor 35 (GPR35) and the aryl hydrocarbon receptor (AhR) [21]. We speculate that the immunomodulatory properties of kynurenic acid may contribute, in part, to the resolution of inflammation observed in the present study.

The conditioning of the colon afforded by walnut supplementation may also be associated with the ellagitannins, a group of complex phytochemicals commonly found in walnuts and other natural products that give rise within the gut to ellagic acid and the urolithins, a potent family of antioxidants [1]. In fact, it has been shown that ellagic acid exhibits more potent radical scavenging and metal chelating activities than other well-known antioxidant compounds, including the tocopherols and ascorbic acid [44]. In addition, several studies have shown that administration of ellagic acids or pomegranate polyphenolics significantly attenuated DSS-induced colitis [45,46,47]. Furthermore, free ellagic acid is metabolized by the gut microbiome to the urolithins [48], a large group of molecules that have been extensively studied for their antioxidant, anti-inflammatory and anti-cancer activities [49,50,51,52]. In fact, a recent study by Singh et al. [53] showed that urolithin A and its potent synthetic analogue protected from 2,4,6-trinitrobenzene sulphonic acid (TNBS)-induced or DSS-induced colitis through enhancement of gut barrier function by activating the nuclear factor erythroid 2-related factor 2 (Nrf2) pathway. In the present study, we found a modest, although non-significant trend towards increased levels of urolithin A in the colons of mice treated with 14% walnuts (C13H8O4; 1.77-fold; *p* = 0.630; data not shown). Based upon these results, we believe that further targeted analysis of ellagic acid-derived metabolites may be warranted in order to better define the potential role of the urolithins in the maintenance of colon health, particularly with respect to ulcerative colitis. 

## 5. Conclusions

In summary, this study has shown that dietary supplementation of walnuts has the ability to protect mice against DSS-induced experimental colitis. The beneficial effects of dietary walnut consumption may be due in part to sustained alterations to the tissue microenvironment present within the colonic mucosa. A diet enriched in walnuts may shift the overall metabolic state of the colon towards one that is capable of resisting the ulcerogenic actions of DSS-induced injury. Our global discovery-based metabolite analyses have shown variability, possibly due to the relatively modest sample size (*n* = 3~10), and further analyses are warranted to validate these initial findings. However, significant changes in metabolites levels present in both fecal samples and colonic mucosa indicate that regular walnut consumption may improve lipid metabolism and enhance the production of antioxidants in the colon. It is reasonable to speculate that walnut consumption has provided the ’at-risk’ colonic mucosa with a more protective milieu which may better withstand subsequent environmental insults.

## Figures and Tables

**Table 1:** Metabolite alterations in fecal samples after a two-week supplementation with walnuts (*n* = 3/group).

Name	Formula	Molecular Weight	Ratio: Day 14/Day 0	*p*-Value: Day 14/Day 0
9-oxo-10(E),12(E)-octadecadienoic acid	C18 H30 O3	294.2195	29.29	0.0001
Corticosterone	C21 H30 O4	346.2144	11.99	0.0894
Docosahexaenoic acid (DHA)	C22 H32 O2	328.2401	8.33	0.0090
Ellagic acid	C14 H6 O8	302.0061	8.25	0.0883
Arachidonic acid (AA)	C20 H32 O2	304.2402	7.42	0.0283
Kynurenic acid	C10 H7 N O3	189.0426	6.39	0.0042
D-(+)-Proline	C5 H9 N O2	115.0634	3.42	0.0078
Spectinomycin	C14 H24 N2 O7	332.1598	3.22	0.0501
Bis(methylbenzylidene)sorbitol	C22 H26 O6	386.1727	2.59	0.0196
N-Acetylserotonin	C12 H14 N2 O2	218.1055	2.44	0.0225
Benzophenone	C13 H10 O	182.0733	2.36	0.0081
Sulcatol	C8 H16 O	128.1204	2.31	0.0999
L-Methionine sulfoxide	C5 H11 N O3 S	165.0460	1.77	0.0898
Dodecyltrimethylammonium	C15 H33 N	227.2614	0.53	0.0310

**Table 2:** Metabolite alterations in colon tissues after a two-week supplementation with walnuts (*n* = 10/group).

Name	Formula	Molecular Weight	Ratio: Day 14/Day 0	*p*-Value: Day 14/Day 0
Olomoucine	C15 H18 N6 O	298.1545	1.73	0.0098
Adenosine 5’-monophosphate	C10 H14 N5 O7 P	347.0628	1.67	0.0951
L-Tyrosine	C9 H11 N O3	181.0741	1.53	0.0823
Adenosine 5’-monophosphate	C10 H14 N5 O7 P	347.0628	1.52	0.0787
S-Adenosylhomocysteine	C14 H20 N6 O5 S	384.1213	1.50	0.0685
Triisopropanolamine	C9 H21 N O3	191.1521	1.38	0.0009
Flurandrenolide	C24 H33 F O6	436.2281	1.35	0.1114
DL-Glutamine	C5 H10 N2 O3	146.0691	1.31	0.0122
D-Serine	C3 H7 N O3	105.0426	1.31	0.1979
Betaine	C5 H11 N O2	117.0789	1.26	0.1157
Bis(4-ethylbenzylidene)sorbitol	C24 H30 O6	414.2040	1.23	0.1477
Bis(methylbenzylidene)sorbitol	C22 H26 O6	386.1726	0.79	0.0069

## Supplementary Materials

The following are available online at https://www.mdpi.com/2072-6643/11/5/1118/s1, Table S1: Macronutrient and fatty acid composition of the TWD diet, Figure S1: Linear-regression analyses of ulceration and restitution.
