# Supplementary material for: Dietary Walnut Supplementation Alters Mucosal Metabolite Profiles During DSS-Induced Colonic Ulceration
Source: Nutrients. 2019 May 20;11(5):1118. doi: 10.3390/nu11051118 (PMC6566840; doi:10.3390/nu11051118)
Supplement: Supplementary file 1 [file nutrients-11-01118-s001.pdf]

Supplementary Table S1. Macronutrient and fatty acid composition of the TWD diet.

Macronutrient	Walnuts (% by weight)			
	**0**	**3.5**	**7**	**14**
Carbohydrates				
Corn starch	230	225	219	211
Maltodextrin	70	70	70	70
Sucrose	261	261	261	261
Cellulose	30	30	29	27
Kcal (% of total)	50	50	50	50
Proteins				
Casein	190	184	178	165
L-Cystine	2.9	2.9	2.9	2.9
Kcal (% of total)	15.5	15.5	15.5	15.5
Fats				
Olive oil	28	24	20	12
Soybean oil	31	27	23	14
Corn oil	17	14	12	7
Lard, pork	28	24	20	12
Beef tallow	25	21	18	11
Anhydrous milk fat	26	31	26	16
Cholesterol	0.4	0.4	0.4	0.4
Walnuts	0	35	70	140
Kcal (% of total)	34.5	34.6	34.9	35.0
Fatty acids				
Oleic acid	64.3	58.3	52.5	40.2
Linoleic acid	33.3	41.9	50.9	67.6
α-Linoleic acid	3.5	6.1	8.9	14.1
Saturated fat	59.7	53.4	43.0	26.1
Kcal from Walnuts (% of total)	0	5.2	10.5	21.4

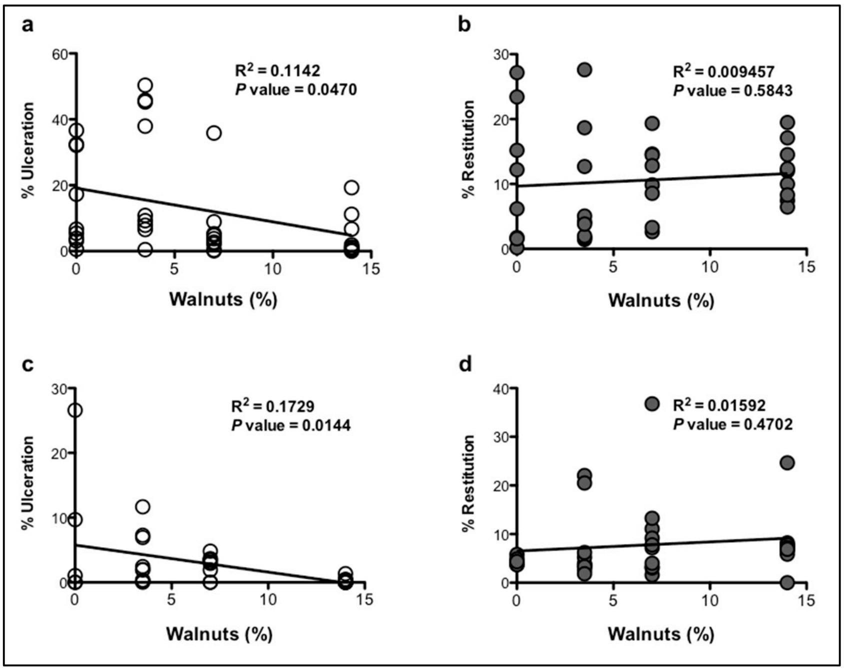

Supplementary Figure S1. Linear-regression analyses of % ulceration (a) and % restitution (b) in DSS-induced acute colitis, and % ulceration (c) and % restitution (d) in the recovery from DSS-induced colitis.
